# Sulfur-Containing Amino Acids, Hydrogen Sulfide, and Sulfur Compounds on Kidney Health and Disease

**DOI:** 10.3390/metabo13060688

**Published:** 2023-05-25

**Authors:** Chih-Jen Chen, Ming-Chou Cheng, Chien-Ning Hsu, You-Lin Tain

**Affiliations:** 1 Department of Pediatrics, Kaohsiung Chang Gung Memorial Hospital, Kaohsiung 833, Taiwan; 2 Department of Pharmacy, Kaohsiung Chang Gung Memorial Hospital, Kaohsiung 833, Taiwan; cnhsu@cgmh.org.tw; 3 School of Pharmacy, Kaohsiung Medical University, Kaohsiung 807, Taiwan; 4 Institute for Translational Research in Biomedicine, Kaohsiung Chang Gung Memorial Hospital, Kaohsiung 833, Taiwan; 5 College of Medicine, Chang Gung University, Taoyuan 333, Taiwan

**Keywords:** hypertension, cysteine, kidney disease, developmental origins of health and disease (DOHaD), hydrogen sulfide, sulfur-containing amino acids, organosulfur compounds

## Abstract

Hydrogen sulfide (H_2_S) plays a decisive role in kidney health and disease. H_2_S can ben synthesized via enzymatic and non-enzymatic pathways, as well as gut microbial origins. Kidney disease can originate in early life induced by various maternal insults throughout the process, namely renal programming. Sulfur-containing amino acids and sulfate are essential in normal pregnancy and fetal development. Dysregulated H_2_S signaling behind renal programming is linked to deficient nitric oxide, oxidative stress, the aberrant renin–angiotensin–aldosterone system, and gut microbiota dysbiosis. In animal models of renal programming, treatment with sulfur-containing amino acids, N-acetylcysteine, H_2_S donors, and organosulfur compounds during gestation and lactation could improve offspring’s renal outcomes. In this review, we summarize current knowledge regarding sulfide/sulfate implicated in pregnancy and kidney development, current evidence supporting the interactions between H_2_S signaling and underlying mechanisms of renal programming, and recent advances in the beneficial actions of sulfide-related interventions on the prevention of kidney disease. Modifying H_2_S signaling is the novel therapeutic and preventive approach to reduce the global burden of kidney disease; however, more work is required to translate this into clinical practice.

## 1. Introduction

Sulfur-containing amino acids cover methionine, cysteine, homocysteine, and taurine. Methionine and cysteine are precursors of glutathione, which play a prominent role in oxidative stress [1]. It is known that oxidative stress is involved in the development of kidney disease [2]. Homocysteine is a non-protein-bound sulfur amino acid implicated in one-carbon metabolism and kidney disease [3]. Taurine, a major end-product of methionine metabolism, is also linked to kidney disease [4]. Additionally, hydrogen sulfide (H_2_S) is endogenously generated from the metabolic pathway of sulfur-containing amino acids and plays a key role in kidney health and disease [5,6].

The main sources of sulfur in the diet are sulfur-containing amino acids and inorganic sulfate. During pregnancy, sulfate is an important nutrient for fetal development [7]. As fetal tissues have a limited capacity to produce sulfate, the source of sulfate for the fetus is mainly dependent on maternal circulation. Apart from the metabolism of sulfur-containing amino acids in pregnant mothers, sulfate can be obtained from sulfur compounds in the maternal diet. Maternal nutrition is the major determinant of fetal morphology and function via a process known as developmental programming [8]. An imbalanced process may provoke renal programming, resulting in kidney disease later in life [9]. This concept is recognized as the developmental origins of health and disease (DOHaD) [10].

According to the DOHaD theory, renal programming processes are able to be reversed or postponed in early life by reprogramming to prevent adulthood kidney disease [9]. Emerging evidence suggests sulfur-containing amino acids, their derivatives, and sulfur compounds may serve as reprogramming strategies to avert kidney disease and promote kidney health [11].

Nowadays, chronic kidney disease (CKD) is still on the rise all over the world [12], despite medical advances made in recent decades. This situation raises questions about whether more attention is required on global kidney health policy, mostly emphasizing early prevention of kidney disease from occurring in early life [12].

**Figure 1:**
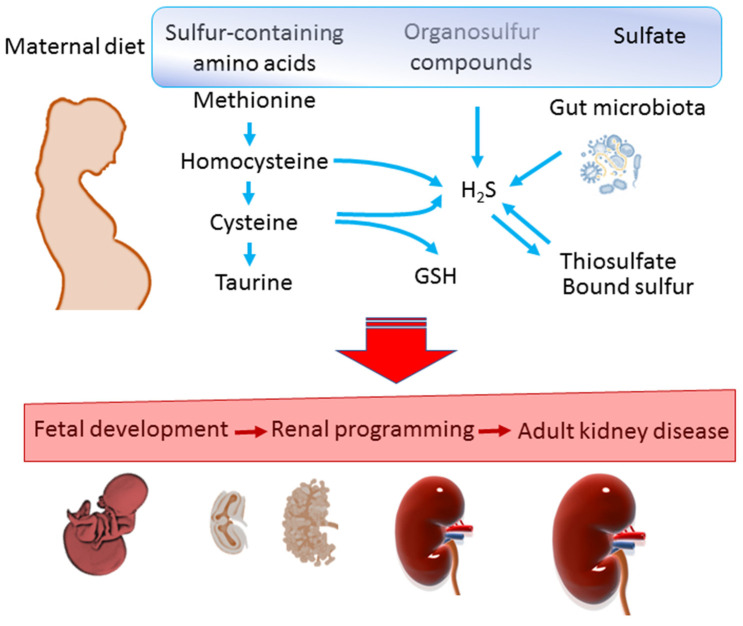
Schematic representation of impact of sulfur-containing amino acids, hydrogen sulfide (H_2_S), and sulfur compounds on kidney health and disease. GSH = glutathione.

Therefore, the purpose of this review is to give an overview of the roles of sulfur-containing amino acids, organosulfur compounds, and sulfate in maternal diets involved in kidney health and disease (Figure 1). Additionally, the uses of sulfide-related interventions as reprogramming interventions to prevent adulthood kidney disease are reviewed.

A literature review was carried out by searching the databases Embase, MEDLINE, and Cochrane Library using keywords relevant to hydrogen sulfide, sulfur-containing amino acid, sulfur, sulfide, organosulfur compound, cysteine, pregnancy, lactation, kidney disease, hypertension, developmental programming, and DOHaD. We found that there are more than 2000 publications related to kidney disease and hydrogen sulfide/sulfur. However, less than 4% belong to DOHaD research. Both positive and negative studies were included. Original articles account for nearly 90% of searchable publications. In total, we screened 71 full-text reports for eligibility. The reference lists of articles were also examined to identify any additional references that would be related to this review.

## 2. Sulfur, Pregnancy, and Fetal Development

Sulfur, a fundamental element, is the third most abundant mineral in our body. The human diet covers a diverse spectrum of inorganic and organic dietary-derived sulfur compounds [13]. Inorganic sulfate (SO_4_^2−^) and sulfites (SO_3_^2−^) are common in foods and water, sulfur-containing amino acids present in meat products, and other organosulfur compounds, such as garlic and onions. The maternal diet is recognized as a critical factor for determining the life-long health of the offspring [8]. Here, we summarize the physiological roles and regulation of sulfur-containing amino acids and sulfate during pregnancy, with a particular focus on their impacts on fetal development.

### 2.1. Sulfur-Containing Amino Acids

During pregnancy, amino acids represent one of the major nutrients for fetal development [8]. A net gain in protein by increasing the demand for amino acids during gestation is required by both the mother and the fetus. These amino acids are derived from the diet, as well as from the turnover of maternal proteins. Sulfur-containing amino acids methionine and cysteine account for approximately 4% of maternal proteins [14].

Methionine is essential for protein synthesis and methylation reactions. In both human and animal studies, low dietary consumption of methionine is related to fetal growth retardation [15,16,17]. In pregnant women, the transsulfuration rate of methionine in early gestation and its transmethylation rate in late gestation were higher than those in nonpregnant women [17]. The high rate of transsulfuration in the first trimester is necessary for supplying cysteine and glutathione to the fetus, suggesting a higher demand for methionine.

One-carbon metabolism maintains the critical function of synthesis of purines, thymidylate, and methylation via multiple methyl transferases driven by the methyl donor s-adenosylmethionine (SAM) [18]. Methionine is a key element of the one-carbon metabolism essential for the transfer of methyl groups from folate to SAM. One-carbon metabolism has profound effects on fetal growth and development, implicating long-term morbidity in the offspring [18]. A high rate of transmethylation during late gestation proposes a greater demand for methyl donors [17]. Although deficit methionine is linked to adverse pregnancy and offspring outcomes, excess dietary methionine may lead to a deficiency of glycine and serine [17]. As any imbalance may worsen the supply of particular amino acids to the fetus, one would need to be extremely cautious in considering maternal methionine supplementation to improve fetal growth and development.

High levels of homocysteine, an intermediate of methionine metabolism, in humans, was associated with adverse pregnancy and fetal outcomes, including spontaneous abortion and premature delivery [19]. Compared to nonpregnant women, plasma concentration of homocysteine was lower in normal pregnancies [17]. Nevertheless, the exact mechanism of homocysteine-lowering during pregnancy remains unclear.

Plasma cysteine levels are lower in the third trimester [20], suggesting cysteine is essential for the fetus. As the fetus is incapable of synthesizing adequate cysteine, transsulfuration in the maternal compartment becomes a great source, other than protein breakdown and diet, of cysteine for the fetus. Cysteine is utilized not only in protein synthesis, but also for the biosynthesis of various sulfur-containing molecules. One important product of cysteine is hydrogen sulfide (H_2_S). H_2_S is a gasotransmitter, which regulates placentation, vascular adaptation, and fetal development during normal pregnancy [21]. In addition, cysteine is the precursor for glutathione synthesis. As glutathione is considered the most abundant endogenous antioxidant, this antioxidant response maintains cellular homeostasis during pregnancy [22].

Taurine, a non-protein amino acid, has long been considered an end-product of the metabolism of sulfur-containing amino acids. Prenatal taurine deficiency induces low birth weights and, in later life, risk of adult disease [23]. Emerging evidence supports the notion that taurine coming from the maternal compartment is crucial for fetal development, resulting in different adult phenotypes [24].

### 2.2. Sulfate

In addition to sulfur-containing amino acids, the major dietary sources of sulfur are inorganic sulfur (sulfate and sulfite) and other forms of organic sulfur present in foods such as onion, garlic, broccoli, etc. Sulfate is present in foods, beverages, and drinking water. In the gut, sulfate-reducing bacteria (SRB) can reduce sulfate to sulfide [25]. Sulfate reduction uses sulfate as the electron acceptor, producing H_2_S as a metabolic end-product [26]. Sulfate is an important nutrient for fetal growth and development [27]. In pregnant women, plasma sulfate concentrations are higher than nonpregnant women and increased by twofold with levels peaking in late gestation [28].

Increased plasma sulfate concentrations originate in increased tubular sulfate reabsorption, which was mediated by increased SLC13A1 expression (encoded for sodium-dependent sulfate transmembrane transporter) in the mother’s kidneys [29]. Sulfate can be actively transported from mother to fetus via the placenta. As sulfate is essential for sulfonation reactions to maintain normal structure and the development of tissues [30], maternal sulfate deficiency is detrimental to fetal development [28]. The findings above provide significant insights into the importance of sulfur-containing amino acids and sulfate in normal pregnancy and fetal development.

### 2.3. Organosulfur Compounds

Organosulfur compounds have shown health-promotion benefits due to their ability to participate in metabolism, cellular functions, and protection of cells from oxidative damage [31]. Vegetables in the Allium and Brassica genus, i.e., garlic, onion, broccoli, cauliflower, cabbage, etc., are good sources of organosulfur compounds.

Organosulfur compounds contain sulfur atoms that are bound with a cyanate group or a carbon atom in a chain or cyclic configuration. Allium species contain diverse bioactive compounds, such as alk(en)yl cysteine sulfoxides; S-allyl cysteine; diallyl; mono-, di-, and tri-sulfides; thiosulfinates; and vinyldithiins. Cruciferous vegetables consist of a diverse group of vegetables containing glucosinolates (GLCs), the precursors of ITCs [32].

So far, only one cohort study has demonstrated that intake of garlic in pregnancy was associated with a decreased risk of spontaneous preterm delivery [33]. Garlic contains diverse organosulfur compounds, such as alliin, dialyllsulfides, and allicin [34]. However, safe doses of organosulfur compounds that could be used by pregnant and lactating women await further clarification.

## 3. Hydrogen Sulfide in Kidney Health and Disease

### 3.1. H_2_S Biosynthesis and Metabolism

**Figure 2:**
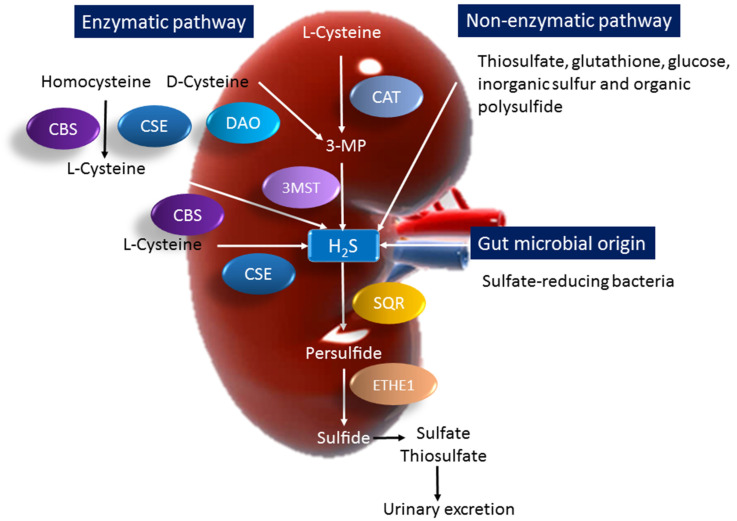
Three major H_2_S synthesis pathways are of enzymatic, non-enzymatic, and gut microbial origin. Cystathionine β-synthase (CBS) or cystathionine γ-lyase (CSE) catalyzes homocysteine to produce l-cysteine. Both CBS and CSE can catalyze l-cysteine to generate H_2_S. 3-mercaptopyruvate sulfurtransferase (3MST) produces H_2_S from 3-mercaptopyruvate (3-MP), which is formed by d-amino acid oxidase (DAO) cysteine aminotransferase (CAT) from d-cysteine and l-cysteine. Another source of endogenous H_2_S is derived from the non-enzymatic synthesis pathway. The other source of H_2_S comes from intestinal bacteria, mainly from sulfate-reducing bacteria. H_2_S is metabolized by sulfide quinone oxidoreductase (SQR) to form persulfide, which can be oxidized by persulfide dioxy-genase (ETHE1) to yield sulfite. Sulfite is converted to sulfate or thiosulfate, which can be excreted into the urine.

H_2_S is a colorless gas with a distinctive smell of rotten eggs. In the 1700s, H_2_S was identified as an environmental toxin [35]. Investigations on the biological effects of H_2_S began around the turn of the 20th century. The production of H_2_S can occur via three origin pathways: enzymatic, non-enzymatic, and bacterial. Figure 2 summarizes enzymatic and non-enzymatic H_2_S synthesis pathways and gut microbial H_2_S production that have been described.

H_2_S is synthesized from l-cysteine via three enzymes, namely cystathionine β-synthase (CBS), cystathionine γ-lyase (CSE), and 3-mercaptopyruvate sulfurtransferase (3-MST) [26]. 3-MST exists in both the mitochondria and cytoplasm, while CBS and CSE are primarily located in the cytosol.

CBS and CSE can decompose l-cysteine and generate H_2_S. They both can also produce H_2_S using other substrates. Homocysteine can be catalyzed by CBS to generate cystathionine, followed by CSE to produce cysteine. 3-MST can also produce H_2_S through a reaction involving the generation of pyruvate from 3-mercaptopyruvate (3-MP), which is provided by cysteine aminotransferase (CAT) and d-amino acid oxidase (DAO). H_2_S can also be derived from d-cysteine by DAO in peroxisomes [36]. Figure 2 illustrates how these enzymes all together regulate physiological H_2_S concentrations in a complex and overlapping manner.

In addition to enzymatic pathways, H_2_S can also be generated via non-enzymatic reactions. Non-enzymatic H_2_S production occurs through thiosulfate, glutathione, glucose, inorganic sulfur, and organic polysulfides (e.g., garlic). Thiosulfate is not only an intermediate of sulfur metabolism, but also a metabolite of H_2_S that can contribute to H_2_S production [37]. Thiosulfate generates H_2_S through a reductive reaction involving pyruvate, which acts as a hydrogen donor. H_2_S can also be formed from glucose, either from phosphogluconate via NADPH oxidase or through glycolysis. Glucose interacts with cysteine, methionine, or homocysteine to yield gaseous sulfur compounds—H_2_S and methanethiol. Additionally, H_2_S is produced through a direct reduction in glutathione and inorganic sulfur. Organic polysulfides can undergo nucleophilic substitution at a sulfur atom, yielding H_2_S and hydropolysulfide [38].

H_2_S can also be produced in the gut by SRB, which obtains energy from the oxidation of organic compounds, reducing sulfate to H_2_S [25]. Approximately 66% of all SRB account for *Desulfovibrio* in the human colon [39]. Other gut bacteria can also generate H_2_S by sulfite reduction, covering species *E. coli*, *Salmonella*, *Enterobacter*, *Bacillus*, *Corynebacterium*, *Klebsiella*, *Rhodococcus*, *Staphylococcus*, etc. [39]. On the other hand, fecal H_2_S can be removed by sulfur-oxidizing bacteria (SOB) via sulfur oxidation. In addition, gut-bacteria-derived H_2_S can also be generated through the fermentation of sulfur-containing amino acids. Large amounts of H_2_S are oxidized by colonocytes into thiosulfate [39].

As shown in Figure 2, H_2_S can be metabolized by a series of enzymatic reactions. Sulfide is oxidized to sulfite in a two-step reaction [40,41]. First, sulfide quinone oxidoreductase (SQR) oxidizes sulfide to generate persulfide [42]. Then, persulfide is oxidized by persulfide dioxygenase (ETHE1) to yield sulfite. As a result, sulfite can be converted to sulfate or thiosulfate by sulfite oxidase (SUOX) and thiosulfate sulfurtransferase (TST), respectively [41]. Sulfide is excreted primarily as sulfate and thiosulfate in the urine.

### 3.2. Biological Function of H_2_S in Kidney

H_2_S has multi-faceted biological functions, including but not limited to antioxidant, anti-inflammation, vasodilatation, mitochondria bioenergetics, metabolic modulation, angiogenesis, and anti-apoptosis [43,44,45]. In the kidney, H_2_S increases the glomerular filtration rate (GFR), inhibits tubular sodium reabsorption, regulates renin release, controls blood pressure (BP), and increases ATP production as a sensor for oxygen [45,46].

All H_2_S-generating enzymes are localized in the kidney. Dual inhibition of CSE and CBS decreased GFR, urinary sodium, and potassium excretion [47]. Conversely, exogenous NaHS administered for 4 weeks increased GFR, urinary sodium excretion, and fractional sodium excretion in spontaneously hypertensive rats (SHRs) [48]. In a two-kidney-one-clip (2K1C) model of renovascular hypertension, NaHS prevented hypertension from accompanying by inhibiting the upregulation of renin mRNA and protein levels in the clipped kidneys [49]. Additionally, H_2_S is able to enhance ATP production and prevent ischemia-reperfusion (IR)-induced kidney damage [50]. Total, cortical, and medullary renal blood flow were reduced in rats with inhibition of CSE and CBS [51]. Meanwhile, renal blood flow can be increased by intrarenal arterial infusion of NaHS [52]. Additionally, a CSE inhibitor decreased blood flow in the renal artery in rats, suggesting CSE-derived H_2_S has a prominent role in regulating renal blood flow and vascular resistance in renal circulation [53].

H_2_S-induced vasodilation has been attributed to several mechanisms, covering the reduction in oxidative stress and inflammation [54], improvement in endothelial function [55], opening of vascular potassium channels [56], augmented NO signaling [57], and activation of vascular endothelial growth factor receptor-2 (VEGFR-2) [58]. The results above reveal that H_2_S is involved in renal physiology and that deficient H_2_S may participate in the pathogenesis of kidney disease.

### 3.3. Impact of H_2_S on Renal Programming

As kidney disease can take its origins in early life via renal programming [59,60], a deeper understanding of how H_2_S impacts renal programming will aid in targeted therapy and the prevention of adult kidney disease. Developing kidneys are vulnerable to adverse environmental stimuli that disrupt fetal development during gestation, resulting in structural changes and functional adaption [59,60]. These risk factors cover imbalanced nutrition, maternal illness, environmental toxins, medication use, etc.

Maternal protein restriction results in harm to kidney development and causes a permanently low nephron endowment [9]. Because the nephron is the basic functional unit of the kidney, a low nephron number can result in glomerular hyperfiltration and compensatory glomerular hypertrophy, and lead to further loss of nephrons [61]. Although methionine and cysteine are essential for protein synthesis [14], whether their deficiencies in the maternal diet are related to low nephron number in renal programming remains unknown. One previous study demonstrated that a maternal methyl-deficient diet caused 938 renal transcripts to be modified and programmed hypertension in adult progeny [62]. In consideration of the view that methionine is part of methyl-donor nutrients [63], its link to H_2_S signaling in renal programming deserves further clarification.

As reviewed elsewhere [59], several maternal illnesses have been related to renal programming, just like hypertensive disorders of pregnancy, preeclampsia, CKD, and diabetes. Interestingly, these maternal diseases are more or less related to abnormal H_2_S signaling [64]. Furthermore, emerging evidence from human evidence and animal models supports the link between environmental toxin exposure during gestation and the developmental programming of kidney disease later in life [65]. It is needless to mention that H_2_S has traditionally been viewed as a toxic gas at high concentrations devoid of any physiological function [66]. Another risk factor for renal programming is medication use. Existing research demonstrates that several drugs administrated during pregnancy may induce renal programming [67]. One example is glucocorticoids. Antenatal glucocorticoid exposure has been relevant to low nephron numbers and renal programming [68]. As glucocorticoids can inhibit CSE expression and H_2_S production [69], glucocorticoid-induced renal programming might be related to abnormal H_2_S signaling. Together, the findings presented above point toward the roles played by abnormal H_2_S signaling in renal programming.

## 4. Sulfide-Related Reprogramming Intervention

The utilization of sulfide-related therapy has been proven to yield benefits in several kidney diseases, such as acute kidney injury [70], CKD [71], diabetic nephropathy [72], drug-induced nephropathy [73], obstructive nephropathy [74], glomerulosclerosis [75], urolithiasis [76], and kidney transplant [77,78]. Still, little attention has been paid to understanding H_2_S signaling pathway during pregnancy and lactation for the prevention of offspring kidney disease. Early intervention, even prior to the disease appearing, is key to preventing the development of adult kidney disease [9]. Studies documenting sulfide-related interventions in animal models for renal reprogramming are summarized in Table 1, restricting interventions to start before the onset of disease [79,80,81,82,83,84,85,86,87,88,89,90,91,92,93].

Table 1 illustrates that rats are the most frequently used animal species. Several developmental programming models have been used to study renal programming, covering the genetic spontaneously hypertensive rat (SHR) model [79,82,83,89,90], maternal CKD model [80,93], maternal high-sugar-diet model [81], prenatal dexamethasone and postnatal high-fat diet [84], N^G^-nitro-l-arginine-methyl-ester (L-NAME) exposure model [85], maternal suramin administration model [86], maternal hypertension [87], maternal nicotine exposure [88], maternal renovascular hypertension model [91], and maternal high-fat-diet model [92]. Hypertension is the major renal-programming-induced adverse outcome being evaluated. Reported sulfide-related interventions include sulfur-containing amino acids, N-acetylcysteine (NAC), H_2_S donors, and organosulfur compounds. It has been reported that sulfide-related interventions have reprogramming effects in rat offspring aged 8 weeks to 8 months, which is in line for adolescents to middle adulthood in humans [94].

### 4.1. Sulfur-Containing Amino Acids

l-cysteine is a substrate for the production of H_2_S. Another substrate for H_2_S generation is d-cysteine [95]. Prior work reported that the d-cysteine pathway is 80-fold greater at H_2_S-producing activity than the l-cysteine pathway in the kidneys [36]. Prior work revealed that high-salt-treated young SHRs supplemented with d- or l-cysteine over a period of 2 weeks were protected against hypertension and kidney damage at 12 weeks old [79]. Another study evaluated whether l- or d-cysteine supplementation in pregnancy can prevent maternal CKD-primed offspring hypertension [80]. Administration of l-cysteine has been shown to enhance renal H_2_S-generating enzyme CBS and CSE expression, increase renal H_2_S-releasing activity, and increase plasma concentration of H_2_S and thiosulfate [80]. Furthermore, d-cysteine supplementation restored CKD-primed reduction in plasma thiosulfate levels, while it had a negligible effect on renal H_2_S-generating enzymes [80].

Another sulfur-containing amino acid used for reprogramming is taurine. Perinatal taurine supplementation was able to protect adult rat offspring against hypertension and kidney dysfunction induced by a maternal high-sugar diet [81]. In SHRs and stroke-prone spontaneously hypertensive rats (SHRSPs), taurine supplementation during pregnancy and lactation had antihypertensive effects on adult offspring [82,83]. Taurine treatment has shown benefits for several kidney diseases, such as diabetic nephropathy [96], renal ischemia/reperfusion injury [97], glomerulonephritis [98], and nephrotic syndrome [99]. Nevertheless, further clarification is needed regarding the reprogramming effects of perinatal taurine supplementation on offspring’s kidney disease.

### 4.2. N-Acetylcysteine

NAC, an N-acetyl derivative of l-cysteine, can also be used to produce H_2_S in experimental studies. Similar to cysteine, early NAC therapy at age 4–12 weeks displayed protection against hypertension in adult SHRs [89]. In addition, administration of NAC during gestation and lactation has been shown to prevent offspring hypertension in several models of renal programming, covering antenatal dexamethasone administration plus post-weaning high-fat diet [85], maternal L-NAME exposure [86], maternal suramin administration [87], maternal hypertension [88], and maternal nicotine exposure [89]. Although several animal models in response to different early-life insults presented protection against hypertension, data are still lacking regarding other reno-protective benefits. It should be noted, however, that NAC is widely used as a pharmacological antioxidant [100].

### 4.3. H_2_S Donors

Inorganic sulfide salts such as sodium hydrosulfide (NaHS) and sodium sulfide (Na_2_S) are the most commonly utilized exogenous H_2_S donors [101,102]. NaHS therapy between 4–8 weeks of age prevented the development of hypertension in 12-week-old SHRs [90]. Another study demonstrated that maternal NaHS therapy protects adult progeny against hypertension in a 2K1C hypertensive model [91].

Inorganic sulfide salts provide direct and prompt release of free H_2_S. As a result, these H_2_S donors might be unsuitable for clinical use due to the rapid increase in H_2_S concentration to supraphysiological concentration. Later on, some organic slow-releasing H_2_S donors are synthesized to better mimic the physiological H_2_S production and overcome this limitation [101,102].

GYY4137 was produced as one of the first slow-releasing H_2_S donors [102]. Even though GYY4137 exerted protective action against hypertension in a CSE inhibition model and an L-NAME-treated SHR model [103,104], organic slow-releasing H_2_S donors have not yet been assessed in terms of their reno-protective effects on renal-programming-induced models. Moreover, thiosulfate can be considered a H_2_S mimetic, which presents the therapeutic potential of sodium thiosulfate for kidney disease [105,106]. We recently found that sodium thiosulfate therapy can produce H_2_S and prevent hypertension concurrently in an adenine-induced CKD model [107]. However, there is little knowledge on whether sodium thiosulfate treatment during gestation and lactation can prevent renal-programming-related adverse offspring’s outcomes.

### 4.4. Organosulfur Compounds

In addition to synthetic H_2_S donors, researchers have focused their attention on natural H_2_S donors. These organosulfur compounds include polysulfides derived from Alliaceae—diallyl di- and tri-sulfide—and GLS-derived ITCs [108].

Garlic-derived organic polysulfides have shown potential benefits as a treatment option in kidney disease and related complications [109,110,111]. Supplementation of garlic oil during gestation and lactation protected against maternal CKD-primed offspring hypertension at 12 weeks of age [92]. In another study examining the reprograming effect of garlic oil in a maternal high-fat model, the rise of BP in 16-week-old offspring was prevented by perinatal garlic oil supplementation [93].

Though interest in exploring the potential therapeutic effects of ITCs has grown with the finding of their ability to release H_2_S [108], their beneficial effect against renal programming has not yet been explored.

### 4.5. Others

The impact of gut-derived H_2_S on renal programming has not been studied, while gut microbiota denotes the greatest source of H_2_S in the body. Abundant SRB and SOB control the generation and degradation of H_2_S in the gut [112]. High concentrations of H_2_S are toxic for the gut epithelium and may contribute to bowel disease. Therapeutic targeting of SRB has been tested to regulate gut-inflammation-related H_2_S production [113]. More research on gut-bacteria-derived H_2_S is required as they may turn into a potential therapeutic target for renal-programming-related diseases.

**Figure 3:**
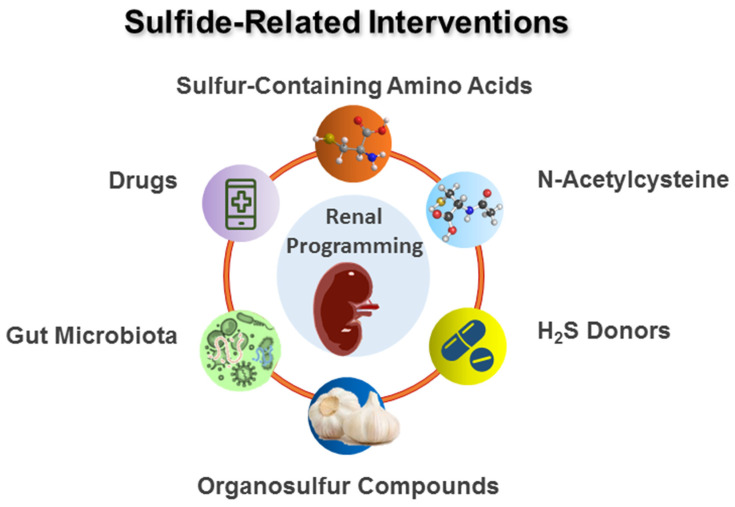
Schema outlining potential sulfide-related interventions used for renal programming.

H_2_S is also regulated by several presently used drugs, such as aspirin, amlodipine, atorvastatin, carvedilol, testosterone, digoxin, metformin, paracetamol, ramipril, vitamin D, and 17β-estradiol [114]. Although metformin was reported to protect maternal high-fructose plus post-weaning high-fat-diet-induced offspring [115], whether it is beneficial for kidney health and related to H_2_S signaling is unclear. It would be interesting to see whether targeting H_2_S-signal-related mechanisms by these drugs would become a practical approach to prevent renal programming for further clinical translations. A summary of potential sulfide-related interventions as reprogramming strategies for renal programming is illustrated in Figure 3.

## 5. Mechanisms behind Protective Actions of H_2_S on Renal Programming

Investigation of the potential mechanisms underlying renal programming has gained increasing attention [9,60]. Currently, the mechanisms accounting for renal programming include deficient NO [116], oxidative stress [2,117], the aberrant renin–angiotensin–aldosterone system (RAAS) [118], and gut microbiota dysbiosis [119,120]. The results of animal experiments indicate that the H_2_S signaling pathway interacts with the abovementioned mechanisms. A summary of the link between H_2_S and other mechanisms involved in renal programming and reprogramming by sulfide-related interventions for the prevention of kidney disease is depicted in Figure 3. Each of these mechanisms are discussed in turn.

### 5.1. Deficient NO

NO, a vasodilator, plays a key role in embryogenesis, regulation of fetoplacental vascular reactivity, and fetal development during gestation [121]. NO deficiency participates in the development of kidney disease, as well as hypertension [122,123]. The critical role of deficient NO implicated in renal programming is supported by several animal models, as we reviewed elsewhere [116]. Renal NO deficiency can be attributed to l-arginine deficiency (the substrate for NOS), diminished NOS activity and abundance, NO inactivation by oxidative stress, and inhibition by asymmetric or symmetric dimethylarginine (ADMA or SDMA) [116].

As revealed in Table 1, renal programming induced by maternal L-NAME administration [85], maternal suramin administration [86], and maternal CKD [92] is associated with impaired NO pathways. Prior work revealed that maternal NO deficiency induced by L-NAME caused renal programming and hypertension in adult offspring [85]. Protective actions of maternal NAC therapy against L-NAME-induced offspring hypertension were associated with increases in renal H_2_S synthesis and H_2_S-producing enzyme expression [85]. In another maternal suramin-induced hypertension model [86], the beneficial effects of NAC were accompanied by increased renal 3MST protein abundance, an increase in plasma glutathione level, and restoration of NO. Another line of evidence for the interplay between H_2_S and NO implications in renal programming was obtained in a maternal CKD model, which showed that the protective effects of perinatal garlic oil supplementation against offspring hypertension coincided with enhanced H_2_S signaling and increased NO bioavailability [92].

Increasing evidence supports the assumption that H_2_S and NO affect not only the production of each other but also the further downstream signaling pathway [124]. H_2_S causes the increase in NO bioavailability in several ways, such as reduction in ADMA [125], activation of eNOS via calcium influx or Akt activation [126,127], diminished cGMP degradation [128], reduction in nitrite [129], and augmenting eNOS activity by S-sulfhydration [130]. Though there is a lot of evidence pointing towards their close connection, additional research is needed to explore the crosstalk between H_2_S and NO in renal programming and reprogramming.

### 5.2. Oxidative Stress

The developing kidney is vulnerable to oxidative damage stress due to the low antioxidant capacity of the fetus [131]. Oxidative stress is a phenomenon caused by an imbalance between oxidants and antioxidants in favor of the oxidants. Oxidative stress and renal programming are intertwined in several animal models, covering maternal CKD [80], prenatal dexamethasone plus post-weaning high-fat diet [84], maternal suramin administration [84], and maternal nicotine exposure [88], as listed in Table 1.

The role of H_2_S as an antioxidant in the renal oxidative stress response has been widely noted [45]. This occurs through scavenging ROS; increasing antioxidants, such as glutathione, superoxide dismutase (SOD), and nuclear factor E2-related factor 2 (Nrf2); and downregulating ROS-generating enzymes, such as NADPH oxidase [45].

Accumulative evidence has supported the reprogramming effects of perinatal antioxidant therapy on renal programming and how this may prevent adult-onset kidney disease [132]. In a maternal CKD model [80], the protective effect of both l- and d-cysteine against hypertension in adult rat offspring was accompanied by the reduction in renal oxidative damage. Additionally, the utilization of NAC during gestation and lactation was reported to reprogram hypertension and reduce renal oxidative stress concurrently in animal models of prenatal dexamethasone plus post-weaning high-fat diet [84], maternal suramin administration [86], and maternal nicotine exposure [88].

Although some sulfide-related interventions have previously been shown to counterbalance oxidative stress to protect offspring against renal programming, whether the antioxidant property of H_2_S has the greatest impact in preserving kidney health compared to other mechanisms still awaits further elucidation.

### 5.3. Aberrant RAAS

The RAAS is a key hormone cascade regulating BP and the renal system [133]. There are two pathways of the RAAS: classic and non-classic systems. The classic RAAS is composed of angiotensin-converting enzyme (ACE), angiotensin (Ang) II, and angiotensin type 1 receptor (AT1R). On the other hand, the ACE2–angiotensin (1–7)–Mas receptor pathway is a counter-regulatory RAAS system that offsets the harmful effects of Ang II signaling.

H_2_S is known to influence several elements of the RAAS system, including decreasing the release of renin [134], inhibiting ACE activity [135], and reducing AT1R expression [136]. Conversely, pharmacological inhibition of CSE leads to increases in ACE and AT1R expression [137]. Taken together, existing evidence indicates that H_2_S suppresses the biological effects of the classic RAAS.

During kidney development, RAAS genes are highly expressed and have a transient biphasic response with the downregulation of the classic RAAS in neonates that becomes normalized over time [60,138]. Various early-life environmental insults interrupt this normalization and improperly initiate the classic RAAS, resulting in kidney disease and hypertension later in life [118]. Meanwhile, early blockade of the classic RAAS has revealed benefits against offspring hypertension in several models of renal programming [118]. These observations can provide support for the role of aberrant RAAS in renal programming.

In SHR, downregulated H_2_S-generating enzymes and low concentrations of H_2_S were reported in hypertensive rats, accompanied by activation of the classic RAAS [90]. NaHS treatment protected against hypertension coincided with the downregulation of classic-RAAS-related gene expression [87]. In a maternal renovascular hypertensive model, NaHS treatment also prevented the rise in BP in adult offspring, together with reducing the AT1R protein level [136]. Although the beneficial action of H_2_S has been linked to the activation of non-classic RAAS systems [139], no information currently exists regarding whether the reprogramming effect of H_2_S on renal programming is due to non-classic RAAS.

### 5.4. Gut Microbiota Dysbiosis

Gut microbiota have been implicated in the regulation of the absorption and metabolism of dietary nutrients that influence human health and disease [140]. The bidirectional link between the gut microbiota and kidney disease is termed the gut–kidney axis [141]. Gut–kidney axis dysfunction due to gut microbiota dysbiosis is implicated in kidney disease [119,120]. So far, some mechanisms underlying gut microbiota dysbiosis have been connected to kidney disease, including increases in trimethylamine-N-oxide (TMAO), alterations of short-chain fatty acids (SCFAs), and increases in tryptophan-derived uremic toxins [142,143]. Kidney disease can be treated or modified through agents that modulate the gut microbes and their metabolites, including prebiotics, probiotics, postbiotics, etc. [142,143,144].

Maternal nutritional insults alter gut microbiota composition and function, resulting in an increased risk of developing adult diseases [145]. Nevertheless, whether early gut-microbiota-targeted therapy may serve as a reprogramming strategy to prevent the developmental programming of kidney disease remains largely unknown [120]. In a maternal CKD model, l-cysteine supplementation protection against offspring hypertension is related to reshaping the gut microbiome [80]. Tryptophan metabolites, such as indole derivatives, are well-known uremic toxins [146]. The beneficial actions of l-cysteine supplementation are associated with the depletion of indole-producing genera *Akkermansia* and *Alistipes*, reduction in several indole metabolites, and enhancement of beneficial genera *Butyricicoccus* and *Oscillibacter*.

Another study reported that maternal NAC therapy protects male SHR progeny against hypertension and is connected to increased fecal thiosulfate levels and alterations of gut microbiota compositions [87]. NAC therapy increased the abundance of genus *Bifidobacterium* and its related phylum *Actinobacteria*, a common SOB [147]. Given that NAC enhanced *Actinobacteria* abundance and fecal thiosulfate levels concurrently, and that SOB can oxidize H_2_S to thiosulfate, it is possible that the beneficial actions of NAC are relevant to increased SOB and their derived thiosulfate production.

Maternal garlic oil supplementation prevented maternal CKD, and high-fat-diet-primed offspring hypertension was also relevant to modifications in gut microbiota [92,93]. Apart from the increased abundance of the genus *Lactobacillus*, a known probiotic, garlic oil supplementation increases plasma concentrations of SCFAs [93].

Together, these results establish a tight connection between H_2_S and other important mechanisms behind renal programming. The advantageous effects of sulfide-related therapy on renal programming are associated widely with deficient NO, oxidative stress, aberrant RAAS, and gut microbiota dysbiosis. Nevertheless, additional research is required to gain an understanding of how H_2_S may play a major role in mediating other mechanisms to develop a specific reprogramming strategy for the prevention of kidney disease.

## 6. Conclusions and Perspectives

The kidney is a major contributor to overall endogenous H_2_S generation, and H_2_S appears to play a significant role in kidney health and disease. Similar to adult kidney disease, deficient H_2_S is present in early life, resulting in renal programming. The dysregulated H_2_S signaling underlying renal programming is connected to deficient NO, oxidative stress, aberrant RAAS, and gut microbiota dysbiosis. The importance of sulfide-related interventions during gestation and lactation in reprogramming kidney disease is highlighted by the observations that sulfur-containing amino acids, NAC, H_2_S donors, and organosulfur compounds prevent offspring’s renal adverse outcomes in a variety of animal models.

One crucial aspect to consider is that research carried out so far has mainly focused on H_2_S-releasing drugs. However, how gut-bacteria-derived H_2_S participate in renal programming is largely unclear. Whether gut-derived H_2_S is beneficial for kidney health and whether gut-microbiota-targeted therapies may alter SRB/SOB to affect gut-derived H_2_S seems worthy of investigation. Another important aspect of H_2_S biology that remains unexplored is the identity of the molecular targets of H_2_S in the kidney, especially during kidney development. As H_2_S can impact multiple proteins and signaling pathways via sulfhydration in the kidney [130], it may act through the crosstalk with other molecular mechanisms to induce renal programming. It should be kept in mind that H_2_S at supraphysiologic concentrations is toxic. Clinical trials should be performed to test whether promising data from animal studies can be translated into human therapies. Attention needs to be paid to accurately monitor the concentration of H_2_S in vivo, to increase the efficiency of sulfide-related interventions, and improve kidney-targeting properties.

## Figures and Tables

**Table 1:** Summary of sulfide-related interventions utilized as reprogramming strategies in animal models of renal programming.

Sulfide-Related Intervention	Animal Models	Species/ Gender	Age at Evaluation	Reprogramming Effects	Ref.
Sulfur-containing amino acids					
l-cysteine (8 mmol/kg/day) from 4 to 6 weeks of age	High-salt SHR	SHR/M	12 weeks	Prevented hypertension and kidney damage	[79]
d-cysteine (8 mmol/kg/day) from 4 to 6 weeks of age	High-salt SHR	SHR/M	12 weeks	Prevented hypertension and kidney damage	[79]
l-cysteine (8 mmol/kg/day) during gestation	Maternal CKD	SD rat/M	12 weeks	Prevented hypertension and reduced renal oxidative stress	[80]
d-cysteine (8 mmol/kg/day) during gestation	Maternal CKD	SD rat/M	12 weeks	Prevented hypertension and reduced renal oxidative stress	[80]
3% taurine in drinking water during gestation and lactation	Maternal high-sugar diet	SD rat/F	8 weeks	Prevented hypertension and improved renal function	[81]
3% taurine in drinking water during gestation and lactation	Genetic hypertension model	SHR/M	22 weeks	Prevented hypertension	[82]
5% taurine in drinking water during gestation and lactation	Genetic hypertension model	SHRSP/M	3 months	Prevented hypertension	[83]
N-acetylcysteine					
1% NAC in drinking water during gestation and lactation	Prenatal dexamethasone plus post-weaning high-fat diet	SD rat/M	12 weeks	Prevented hypertension and reduced renal oxidative stress	[84]
1% NAC in drinking water during gestation and lactation	Maternal L-NAME exposure	SD rat/M	12 weeks	Prevented hypertension and altered renal transcriptome	[85]
1% NAC in drinking water during gestation and lactation	Maternal suramin administration	SD rat/M	12 weeks	Prevented hypertension	[86]
1% NAC in drinking water during gestation and lactation	Maternal hypertension	SHR rat/M	12 weeks	Prevented hypertension	[87]
NAC (500 mg/kg/day) in drinking water from gestational day 4 to postnatal day 10	Maternal nicotine exposure	SD rat/M	8 months	Prevented hypertension and reduced oxidative stress	[88]
2% NAC in drinking water from 4 to 12 weeks of age	Genetic hypertension model	SHR/M	12 weeks	Prevented hypertension	[89]
H_2_S donors					
NaHS (14 μmol/kg/day) daily intraperitoneal injection from 4 to 8 weeks of age	Genetic hypertension model	SHR/M	12 weeks	Prevented hypertension	[90]
NaHS (56 μmol/kg/day) daily intraperitoneal injection during gestation and lactation	2-kidney, 1-clip renovascular hypertension model	SD rat/M and F	16 weeks	Prevented hypertension	[91]
Organosulfur compounds					
Garlic oil (100 mg/kg/day) during gestation and lactation	Maternal CKD	SD rat/M	12 weeks	Prevented hypertension	[92]
Garlic oil (100 mg/kg/day) during gestation and lactation	Maternal high-fat diet	SD rat/M	16 weeks	Prevented hypertension	[93]

NAC = N-acetylcysteine. NaHS = sodium hydrosulfide. CKD = chronic kidney disease. L-NAME = N^G^-nitro-l-arginine-methyl ester. M = male. F = female. SHR = spontaneously hypertensive rat. SD = Sprague–Dawley.
